# Prosthetic joint infections caused by *Mycobacterium avium* complex: a series of five cases

**DOI:** 10.5194/jbji-7-137-2022

**Published:** 2022-07-06

**Authors:** Katharine Dobos, Gina A. Suh, Aaron J. Tande, Shanthi Kappagoda

**Affiliations:** 1 Division of Infectious Diseases and Geographic Medicine, Stanford School of Medicine, Stanford, CA 94304, USA; 2 Division of Infectious Diseases, Department of Medicine, Mayo Clinic, Rochester, MN 55902, USA

## Abstract

Prosthetic joint infection (PJI) due to *Mycobacterium avium* complex (MAC) is a rare entity. There is limited guidance on management strategies and outcomes. In this paper, we describe the demographics, comorbidities, and clinical course of five patients at two academic institutions, constituting the largest series described to date.

## 1 Introduction

*Mycobacterium avium* complex (MAC) historically represents a rare cause of prosthetic joint infection (PJI), although it remains an important pathogen to consider in routine clinical practice. Patients with immune suppression have improved survivorship and clinical stability enabling them to pursue elective orthopedic interventions, and molecular laboratory techniques have advanced to identify atypical and fastidious pathogens. Guidelines for treatment of pulmonary nontuberculous mycobacteria and PJI provide little information on how to manage PJIs caused by MAC (Daley et al., 2020; Osmon et al., 2013). Among case reports published in the literature, all of the patients reported have a comorbid immune-suppressing condition, including treated rheumatoid arthritis (RA) (Ingraham et al., 2017; Sigler and Newman, 2019), solid organ transplantation (Gupta and Clauss, 2009), hematologic malignancy (Sixt et al., 2020; Tan et al., 2016), and HIV/AIDS (McLaughlin et al., 1994). Here, we present a case series of five patients treated at two institutions with PJI caused by MAC.

## 2 Methods

After institutional review board (IRB) approval or exemption, we reviewed our electronic health records to extract information on comorbidities, diagnosis, surgical and medical therapy, and outcomes for patients with PJI caused by MAC who were treated at our respective institutions. Cases of MAC PJI between 1 January 2001 and 1 June 2021 were identified. All patients over the age of 18 years who underwent treatment for MAC PJI at our institutions were included. Medical and surgical therapies were not standardized and were performed at the discretion of the treating medical/surgical teams. The patient outcomes were followed until documentation of patient death, clinical cure, or failure to follow up at our institution. PJI was diagnosed based on provider clinical judgment at the time of intervention and was retrospectively confirmed using Musculoskeletal Infection Society (MSIS) 2011 criteria (Workgroup Convened by the MSIS, 2011). In one case, given the relative paucity of pathology and cultures available from the prosthetic joint, the diagnosis was made based on a single positive periprosthetic culture with characteristic gross operative findings combined with positive cultures from other sites in the context of disseminated infection.

## 3 Results

The most common comorbidity among patients in our series was RA on therapy, diagnosed in three of the five patients in the series (see Table 1). Four of the five patients met MSIS 2011 (Workgroup Convened by the MSIS, 2011) criteria for PJI based on the presence of two or more positive cultures obtained from the affected prosthetic joint (see Table 2). However, we note that patient no. 5 presented with abnormal joint aspirate parameters, which may have been impacted by background hematologic derangements, and elevated inflammatory markers suspected to be confounded by active hematologic malignancy. Patient no. 4 did not clearly meet MSIS criteria but was diagnosed by means of a single positive joint aspirate culture and compelling clinical symptoms in the context of confirmed disseminated MAC.

**Table 1:** Demographic data related to five cases of MAC PJI.

Patient	Age ${}^{a}$ /	Site	Risk factors for	Clinical presentation/Concurrent MAC pulmonary		Age of prosthesis ${}^{b}$	ESR (mm h ${}^{-1}$ ) at	CRP (mg dL ${}^{-1}$ ) at	Number of positive
	Sex		MAC infection	infection or disseminated disease			time of diagnosis	time of diagnosis	cultures for MAC
1	70/M	L knee	None apparent	Joint pain after initial knee replacement led to revision for presumed aseptic loosening 4 years after index surgery, followed by second revision with liner exchange 6 years after index surgery. Persistent symptoms with negative cultures and inflammatory cell counts (no AFB cultures sent) prompted explantation and spacer placement 8 years after index surgery: one intraoperative culture was positive for MAC, felt to be a contaminant, and not treated. Patient was referred to our institution and had spacer exchange 9 years following index surgery with isolation of MAC on all three intraoperative cultures prompting treatment. Patient was treated for MAC for 8 months prior to having reimplantation done. Patient completed an additional 8 months of MAC therapy after reimplantation.	N	9 years from index surgery; 4 monthsfrom last revision	11	0.7	One intraoperative(thought to be a contaminant), one preoperativeaspiration, three of three intraoperative
2	67/F	R hip	RA treated with methotrexate and leflunomide, prior prednisone use remotely	Patient presented with 3 years of hip pain after a fall. ESR and CRP were elevated, and the aspiration sent to rule out PJI grew MAC (fluid obtained was of insufficient quantity for cell count). Due to concern that this could be a contaminant, the aspiration was repeated 2 months later and again grew MAC. Resection arthroplasty was done 2 months later with MAC on intraoperative AFB culture.	N	26 years from index surgery; 19 years from last revision	80	4.6	One preoperative aspiration, one of one intraoperative
3	74/M	R knee	RA and OA, prior prednisone use discontinued after colonic perforation approx. 1 month after MAC diagnosis	Patient underwent index R TKA at another institution with poor postoperative recovery. Aspiration done 3 months after index surgery showed WBC > 25 000, culture negative (AFB not sent). MRI of the knee 5 months after index surgery showed a loculated cystic mass, and repeat aspiration of the knee and the cyst grew MAC at this time. At the time of resection arthroplasty, 9 months from index TKA, intraoperative cultures grew MAC in two of two AFB cultures and *Actinomyces neuii* in two of five aerobic cultures. Patient was treated for MAC for 8 months prior to having reimplantation done and completed an additional 6 months of MAC therapy after reimplantation.	N	5 months	77 (at time of resection)	8.7 (at time of resection)	One preoperative aspiration, two of two intraoperative
4	77/F	L hip	RA treated with methotrexate and prednisone	PJI occurred as part of a disseminated infection with MAC vertebral osteomyelitis and nodular bronchiectatic pulmonary MAC. Patient underwent lumbar spine surgical debridement and instrumentation. Patient developed L thigh and hip pain after 6 months of MAC therapy and was found to have L hip PJI and retroperitoneal abscess. Repeat surgical cultures from hip explantation and debridement of abscess were positive for MAC. Patient underwent a one-stage exchange and remained on suppressive antibiotics.	Y	15 years	95	4.0	One positive culture from hip aspiration, other positive cultures at sites of dissemination
5	46/F	L knee	SLE treated with prednisone; refractory DLBCL treated with R-CHOP, followed by R-ICE and Vanderbilt chemotherapy ${}^{c}$	Patient presented with L knee pain 1 years after L TKA while being treated for refractory diffuse large B-cell lymphoma. Knee aspirate grew both MAC and *Enterococcus faecium.*	N	1 year	63	46.5	Two aspirations done on separate dates

The abbreviations used in the table are as follows: AFB – acid-fast bacilli; MRI – magnetic resonance imaging; OA – osteoarthritis; PJI – prosthetic joint infection; DLBCL – diffuse large B-cell lymphoma; R-CHOP – rituximab,cyclophosphamide, doxorubicin, vincristine, and prednisone; R-ICE – rituximab, ifosfamide, carboplatin, and etoposide; RA – rheumatoid arthritis; SLE – systemic lupus erythematosus; TKA – total knee arthroplasty;CRP – C-reactive protein; ESR – erythrocyte sedimentation rate; WBC – white blood cell; M – male; F – female; L – left; R – right. ${}^{a}$ At time of diagnosis. ${}^{b}$ At time of PJI diagnosis, relative to time of index implantation(if not otherwise specified). ${}^{c}$ Prednisone, cyclophosphamide, etoposide, doxorubicin, vincristine, bleomycin, methotrexate, and leucovorin.

Among the patients in our series who underwent treatment for MAC PJI, all patients but one underwent combined medical and surgical therapy, as described in Table 2. Surgical therapy included arthroplasty resection in three patients, with two of those patients ultimately undergoing reimplantation arthroplasty. Per surgeon discretion, a spacer was not used in one patient with resection in the context of unclear benefit given preoperative diagnosis of MAC; furthermore, the patient was not anticipated to be a reimplantation candidate due to poor bone stock and surgical risk. One patient underwent debridement with modular component (femoral head and polyethylene liner) exchange. Surgery was deferred due to medical contraindications in one patient. Medical therapy included at least 12–16 months of triple-antibiotic therapy with a rifamycin, ethambutol, and macrolide in all patients, with the extension of therapy to lifelong suppression in the patient with disseminated disease. Clinical cure was achieved in three of five patients at the time of the follow-up, which ranged from 4 months to 7 years. Three of five patients treated had MAC isolates documented to be clarithromycin susceptible at the time of initiation of triple-antibiotic therapy with macrolide backbone. The clarithromycin minimum inhibitory concentration (MIC) was unknown in the remaining two.

**Table 2:** Preoperative synovial fluid analysis and surgical findings of the current series.

	Pre-operative synovial fluid analysis			Surgical findings		Management			
Patient	Cell count anddifferential	AFB smear/culture	Gross findings	Pathology	AFB smear/culture at surgery	Surgical management	Antimicrobial therapy	Outcome	Clarithromycin MIC ${}^{b}$
1	2120 (PMNs 10 %, lymphocytes 24 %, monocytes 66 %)	Negative/MAC	Purulent joint fluid with caseous material	Not sent	Not done/MAC	Spacer exchange followed 8 months later by reimplantation of static weight-bearing spacer with tobramycin, vancomycin, and amphotericin; reimplantation done after 8 months on antibiotic therapy which continued for 8 months post-implantation	Ethambutol for 16 months, rifabutin for 16 months, and azithromycin for 16 months	Clinical cure of MAC PJI; recurrent PJI with *Klebsiella aerogenes* requiring resection arthroplasty 10 months post-reimplantation	1 µg mL ${}^{-1}$ (S)
2	Not done (fluid obtained was of insufficient quantity for cell count)	Negative/MAC	Purulence andcaseating granulomatous material throughout hipjoint and thigh	Necrotizing tissue with foreign body giant cell reaction and chronic inflammation	Negative/MAC	Explantation without spacer placement;no reimplantationperformed	Ethambutol, rifabutin, and azithromycin for 12 months (with a 2-month period during which azithromycin was inadvertently discontinued)	Delayed primary closure requiring return to OR, followed by subtrochanteric femur fracture; clinical cure at the 2-years follow-up (off antibiotics for 1 year)	4 µg mL ${}^{-1}$ (S)
3	Performed 2 months prior to date of first AFB culture obtained, 26 964(PMNs 77 %,lymphocytes 12 %,monocytes 11 %)	Negative/MAC ${}^{a}$	Obvious evidence of infection with abscesses and sinus tracts	Fragments ofsynovium withnon-necrotizing granulomatous inflammation	Negative/ MAC and *Actinomyces neuii*	Two-stage exchange: spacer with tobramycin, vancomycin, and amphotericin; reimplantation done after 8 months on antibiotic therapy which continued for 6 months post-implantation	Ethambutol and azithromycin for 15 months; rifabutin for 1 month, followed by rifampin for 14 months; ampicillin–sulbactam for 2 weeks, followed by penicillin G for 8 weeks, followed by amoxicillin–clavulanate for 12 months (for *Actinomyces*)	Clinical cure at the 2-year follow-up (off antibiotics for 10 months)	1 µg mL ${}^{-1}$ (S)
4	Not done	Not done	Joint capsule, soft tissue and bone were friable	Not sent	Negative/ MAC	Revision with exchange of the head and liner in the acetabular cup	Ethambutol for 7 years; rifampin for 21 months, followed by rifabutin for 6 years (lower MIC); clarithromycin for 2 weeks(GI intolerance), followed by azithromycin for 7 years ; additional therapy – linezolid for 2 months (stopped with increasing MIC); moxifloxacin for 18 months (added when clinically failing, discontinued after clinical improvement)	Died 7 years after diagnosis while remaining on suppressive antibiotics; cause of death was viral pneumonia with hypercarbic respiratory failure	Unknown
5	5764 (PMNs 7 %, lymphocytes 10 %, monocytes 83 %)	Negative/MAC and *Enterococcus faecium*; repeat aspiration + MAC	NA	NA	NA	Surgery not done due to neutropenia and thrombocytopenia	Azithromycin and ethambutol for 4 months (until death), rifabutin (switched to rifampin after 1 month due to rifabutin shortage) for 4 months (until death)	Died 4 months after diagnosis due to refractory diffuse large B-cell lymphoma with central nervous system involvement	Unknown

The abbreviations used in the table are as follows: PMNs – polymorphonuclear leukocytes, MIC – minimum inhibitory concentration, S – susceptible, GI – gastrointestinal, and NA – not available. ${}^{a}$ Identified on re-aspiration 2 months following initial aspiration for cell count/differential. ${}^{b}$ Using broth dilution method.

## 4 Discussion

To date, the literature has described six cases of individuals who experienced PJI due to MAC, all of whom were considered to be immunosuppressed (Gupta and Clauss, 2009; Ingraham et al., 2017; McLaughlin et al., 1994; Sigler and Newman, 2019; Sixt et al., 2020; Tan et al., 2016). In our case series, the majority of patients (three out of five) were undergoing treatment for RA, one patient had a history of systemic lupus erythematosus (SLE) and subsequent hematologic malignancy, and one patient did not have a documented immunosuppressing risk factor for the development of MAC PJI. This raises the question of whether this patient possessed an undiagnosed immunologic deficiency which increased the risk of MAC infection, and similar cases may benefit from a formal immunology evaluation. Alternatively, there may have been a unique exposure with high inoculum load that placed this patient at risk of infection which was not captured in the medical history. Another interesting finding in this series is the absence of documented disseminated or multi-organ disease among the patients with MAC PJIs; only one of the five patients was noted to have concurrent MAC pulmonary nodular bronchiectatic (as well as native vertebral) disease. There was a wide range of prosthesis ages at the time of MAC PJI diagnosis, with a minimum of 4 months and a maximum of 26 years postoperatively, which suggests that the mechanism of inoculation may have been different among the patients described, with earlier manifestation suggesting possible introduction at time of surgery and later manifestations potentially related to hematogenous or direct traumatic seeding after an environmental exposure. In three of the five cases, MAC was the sole pathogen isolated in the orthopedic cultures. While this could raise suspicion for MAC being a contaminant rather than true pathogen, four of the patients in this series had multiple periprosthetic cultures positive for MAC (except for the patient with disseminated infection, who had a single positive culture from the affected prosthetic joint but numerous positive cultures from other sites of infection).

There was a range of C-reactive protein (CRP) and erythrocyte sedimentation rate (ESR) values as well as joint fluid cell counts at the time of diagnosis, although the limited availability of a full range of data from joint aspirations preoperatively (only available for one of the five patients) makes extrapolation of the significance of this finding challenging. Of the patients who did have documented cell counts with their aspirations, only one out of the three had an elevated synovial fluid white blood cell (WBC) count and elevated PMN (polymorphonuclear leukocyte) percentage according to the 2011 MSIS criteria. Our case series shows that there can be heterogeneity in inflammatory markers and joint aspirate parameters among patients ultimately diagnosed with MAC PJI, which could be related to the severity or acuity of infection, underlying host hematologic and immunologic factors, and concurrent use of immunomodulating therapies. Outcomes among patients in our series were generally positive, with a combination of surgical management as well as prolonged (12–16 month) combination antimycobacterial therapy similar in duration to that recommended for pulmonary disease (Daley et al., 2020). However, one patient experienced another episode of PJI due to an unrelated organism after apparent cure of MAC PJI, another patient later died of an unrelated cause while remaining on suppressive MAC therapy due to disseminated disease, and yet another patient succumbed to their hematologic malignancy. This would, unsurprisingly, suggest that there is a high risk of morbidity and mortality in patients with MAC PJI due to comorbidities with MAC infection reflecting underlying host factors.

As techniques for making the diagnosis of PJI evolve, including the increasing use of molecular methods for pathogen diagnosis (which may improve the rapidity of the diagnosis of slower-growing pathogens such as MAC), we anticipate that there will be an increasing number of cases of MAC PJI described in the literature. We hope this will facilitate the development of a more cohesive strategy to identify patients at greatest risk of MAC PJI early in the disease course.

Clinicians should be aware of the potential for MAC to cause PJI in a variety of clinical contexts, and we would recommend sending AFB (acid-fast bacilli) cultures as part of the PJI workup among patients with RA or other immune-suppressing conditions as well as negative prior routine bacterial cultures.

## Data Availability

Data for this project were obtained from the patients' electronic medical records. No additional data are available.
